# Global transcriptional profiling of the toxic dinoflagellate *Alexandrium fundyense *using Massively Parallel Signature Sequencing

**DOI:** 10.1186/1471-2164-7-88

**Published:** 2006-04-25

**Authors:** Deana L Erdner, Donald M Anderson

**Affiliations:** 1 Marine Science Institute, University of Texas at Austin, Port Aransas, Texas 78373, USA; 2 Biology Department, Woods Hole Oceanographic Institution, Woods Hole MA 02543, USA

## Abstract

**Background:**

Dinoflagellates are one of the most important classes of marine and freshwater algae, notable both for their functional diversity and ecological significance. They occur naturally as free-living cells, as endosymbionts of marine invertebrates and are well known for their involvement in "red tides". Dinoflagellates are also notable for their unusual genome content and structure, which suggests that the organization and regulation of dinoflagellate genes may be very different from that of most eukaryotes. To investigate the content and regulation of the dinoflagellate genome, we performed a global analysis of the transcriptome of the toxic dinoflagellate *Alexandrium fundyense *under nitrate- and phosphate-limited conditions using Massively Parallel Signature Sequencing (MPSS).

**Results:**

Data from the two MPSS libraries showed that the number of unique signatures found in *A. fundyense *cells is similar to that of humans and *Arabidopsis thaliana*, two eukaryotes that have been extensively analyzed using this method. The general distribution, abundance and expression patterns of the *A. fundyense *signatures were also quite similar to other eukaryotes, and at least 10% of the *A. fundyense *signatures were differentially expressed between the two conditions. RACE amplification and sequencing of a subset of signatures showed that multiple signatures arose from sequence variants of a single gene. Single signatures also mapped to different sequence variants of the same gene.

**Conclusion:**

The MPSS data presented here provide a quantitative view of the transcriptome and its regulation in these unusual single-celled eukaryotes. The observed signature abundance and distribution in *Alexandrium *is similar to that of other eukaryotes that have been analyzed using MPSS. Results of signature mapping via RACE indicate that many signatures result from sequence variants of individual genes. These data add to the growing body of evidence for widespread gene duplication in dinoflagellates, which would contribute to the transcriptional complexity of these organisms. The MPSS data also demonstrate that a significant number of dinoflagellate mRNAs are transcriptionally regulated, indicating that dinoflagellates commonly employ transcriptional gene regulation along with the post-transcriptional regulation that has been well documented in these organisms.

## Background

Dinoflagellates are a group of single celled algae that compose a highly diversified phylum that displays an amazing range of ecological adaptation. Different species employ autotrophy, heterotrophy or mixotrophy, many are known to be symbiotic or parasitic, and bioluminescence is common. They are found at all latitudes and are often a significant component of marine and freshwater phytoplankton communities. Dinoflagellates are also notable for their unusual genome content and organization (reviewed in [1,2]). Estimates of dinoflagellate DNA content range from 3 to 250 pg·cell^-1 ^[3,4], corresponding to approximately 3000–215,000 Mb (in comparison, the haploid human genome is 3180 Mb and hexaploid *Triticum *wheat is 16,000 Mb). It has been suggested that polyploidy or polyteny may account for this large cellular DNA content [5], but studies of DNA reassociation kinetics do not support this hypothesis. Dinoflagellates have many chromosomes (up to 325) that are permanently condensed and attached to the nuclear envelope during cell division [6]. Dinoflagellates are the only eukaryotes with DNA that contains 5-hydroxymethylmuracil, which replaces 12–70% of the thymidine [7].

The unique physical features of the dinoflagellate chromosomes are likely to affect both gene transcription and regulation. While there is an increasing amount of expressed sequence tag information available for dinoflagellates, very few genes have been well characterized with respect to their gene structure and regulation. The few nuclear genes that have been isolated from genomic DNA seem to uniformly lack typical eukaryotic transcriptional elements (e.g. TATA boxes) and polyadenylation sites [8-10]. Studies of dinoflagellate gene expression indicate that these organisms employ both transcriptional (e.g. *pcp *[11]; *Sahh*, *Map *and *Haf *[12] and post-transcriptional (e.g. *lbp *[13]; GAPDH [14]) regulation, with the iron superoxide dismutase of *Lingulodinium polyedrum *exhibiting both modes, depending upon the stimulus [15]. Recent results from microarray analysis of the dinoflagellate *Pyrocystis lunula *indicate that approximately 3% of the transcripts included on the array exhibit transcriptional regulation [16,17].

Together, all of the above data suggests that the organization and regulation of dinoflagellate genes may be different from that of most other eukaryotes. Early microscopic observations of the unusual dinoflagellate nuclear structure led to the hypothesis that dinoflagellates were "mesokaryotes", an intermediate between prokaryotic and eukaryotic microorganisms [18]. However, molecular phylogenetic evidence has since clearly identified them as eukaryotes, and their phylogenetic placement supports Loeblich's (1976) [19] evolutionary interpretation that the unusual properties of dinoflagellate nuclei are derived and not representative of a mesokaryotic ancestral state. As such, our basic knowledge of eukaryotic genetics and gene expression could only be increased by understanding how (and why) dinoflagellates structure their genes and regulate transcription within the sheer quantities of DNA in their cells. To date, most of the data of gene regulation mechanisms in dinoflagellates has emerged sporadically, from studies of specific genes that are of interest for a particular function. The advent of genomic technologies, in particular global gene expression profiling methods, provides the ability to learn about many genes or transcripts simultaneously, even in uncharacterized systems like dinoflagellates. The application of transcriptional profiling to dinoflagellates, in conjunction with laboratory-based gene characterization, holds tremendous potential for understanding gene regulation in this unique and understudied group. In addition, the availability of broad-based gene expression data has the potential to greatly accelerate the pace of research and discovery for dinoflagellates, algae in general and eukaryotic systems as a whole.

This report describes a global and quantitative analysis of the transcriptome of a dinoflagellate. As a model we have used *Alexandrium fundyense*, a species that is capable of producing potent neurotoxins, called saxitoxins, which are the causative agent of paralytic shellfish poisoning. The genus comprises approximately 30 different species that are found worldwide, and 10 of which are known to be toxic and cause so-called "red tides" or harmful algal blooms. This study examined gene expression in nutrient-stressed *Alexandrium *cells using Massively Parallel Signature Sequencing (MPSS), a proprietary technology developed by Solexa, Inc [20]. The method is similar to the well-known Serial Analysis of Gene Expression (SAGE) [21] in that it acquires a short DNA sequence from a defined position in each gene transcript. However, the depth of sampling with MPSS is much greater, with the resulting data set containing at least 1 million, 17-nucleotide 'signature' sequences, making the technology sensitive to genes expressed at low levels.

The MPSS method is "global" in that it provides quantitative expression information for the entire transcriptome. For uncharacterized organisms like dinoflagellates, MPSS can provide a broader view of the transcriptome than microarray expression profiling, which generally includes only a portion of the transcripts present in a cell. Statistical methods for the analysis of quantitative expression data have demonstrated that the MPSS data are robust [22]. Accepting an estimate of 300,000 mRNA molecules in an average eukaryotic cell, MPSS constitutes a three-fold sampling of a single cell, allowing the identification, comparison and quantification of even rare transcripts. The resulting *Alexandrium *MPSS data provide a quantitative assessment of the magnitude of transcriptional regulation in dinoflagellates. Comparison of the *Alexandrium *results to those of other eukaryotes indicates that the distribution and abundance of signature sequences is quite similar in *Alexandrium*, humans and *Arabidopsis*. Finally, identification of MPSS signatures via sequencing provides insight into one mechanism that may contribute to the observed signature number in *Alexandrium*.

## Results

### Physiological status of cells

**Figure 1:**
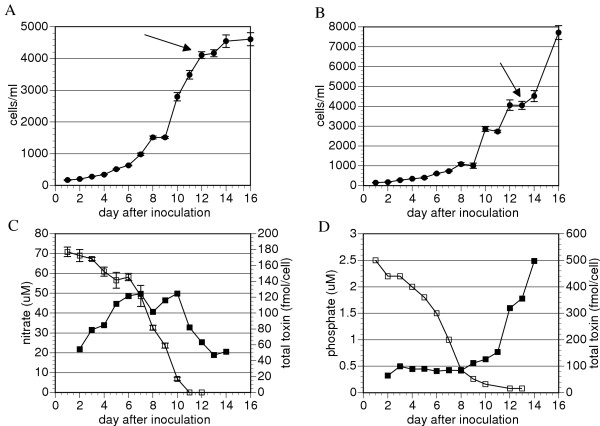
**Growth, nutrient and toxin characteristics of nutrient-limited cultures**. Cell densities were recorded daily in nitrate- (A) and phosphate- (B) limited cultures of A. fundyense. Arrows indicate the day that samples were collected for MPSS analysis. After harvest, the cultures were enriched with the limiting nutrient and changes in cell number were monitored. Nutrient concentrations (open symbols) and toxin per cell (closed symbols) were also monitored daily in the nitrate- (C) and phosphate- (D) limited cultures.

Cells were harvested for analysis at the point at which growth began to slow, at the transition between late logarithmic and stationary phases of growth (Figure 1A and 1B). At this time, the limiting nutrient had been depleted for at least one day (Figure 1C and 1D), resulting in nutrient starvation. The nutrient status of the cells was also reflected in their cellular toxin content (Figure 1C and 1D), which was decreasing in the N-starved culture (Figure 1C) and increasing in the P-starved culture (Figure 1D). This increment or decrement in toxin content under P- or N-starved conditions, respectively, has been well documented for this organism (e.g. [23]). At the time of harvest, P-starved cells contained approximately ten times more toxin than the N-starved cells.

### MPSS signature abundances

An MPSS library was generated for each of the nutrient conditions (N/40 and P/40). Four sequencing runs were performed for each sample, resulting in 2,259,219 successful sequences for the N/40 library and 1,501,972 successful sequences for the P/40 library (Table 1). From the complete sequence sets, 44,779 distinct signatures were observed in the N/40 library, with 27,722 distinct signatures in the P/40 library. The signature sets were filtered to remove any signatures that were 1) not reliable: observed in only one sequencing run, and 2) not significant: never observed at or above 4 tpm in either library. Of the 55,472 unique signatures in the complete sequence set, 33% (18,435 signatures) did not meet the reliability criterion. The significance filter removed an additional 6119 signatures. After filtering, the final set of "reliable and significant" signatures comprised 27,217 signatures for N/40 and 20,161 signatures for P/40. For the purposes of this analysis, we are concerned only with these reliable and significant signatures and will hereafter refer to them simply as "signatures", reserving the term significant to refer to the statistical analysis of differences in gene expression. When the data from the two libraries was combined, a total of 30,917 non-redundant signatures were observed. Of these, 10,756 were not observed in the P/40 library and 3,700 were not present in the N/40 library.

### MPSS signature distributions

**Figure 2:**
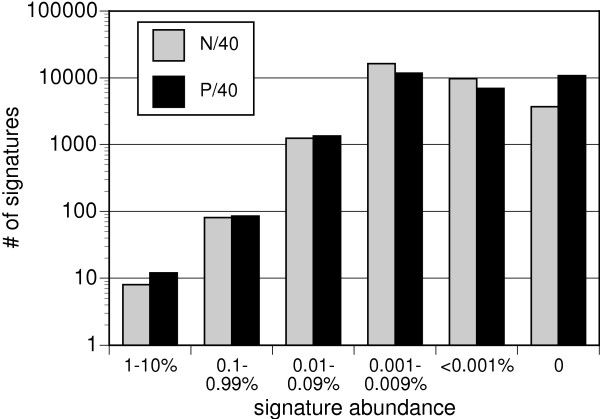
**Signature abundance distributions**. The abundance of each signature is given as a percentage of total signatures in the N/40 (grey bars) and P/40 (black bars) libraries. For example, 1% abundance indicates a signature that is present 10,000 times in each 1,000,000 sequences (10,000 tpm).

The distribution of signature abundances across both libraries was generally quite similar (Figure 2). Only 0.5% of all signatures – 8 signatures in the N/40 library and 12 signatures in the P/40 library – were present at greater than 10,000 tpm (i.e. ≥1% of all sequences). The three most abundant signatures in both libraries constituted 8.9%, 6.2% and 5.6% of total sequences (20.7% combined); all other high abundance signatures were less than 2.5% of sequences. Of the signatures present in a given library, the vast majority, 93% of P/40 and 95% of N/40, were found at less than 0.01% abundance (less than 100 tpm). Of the total signatures, 12% of them were not found in the N/40 library, whereas almost 3 times as many (34.8%) were absent from the P/40 library.

### Differential expression of MPSS signatures

**Figure 3:**
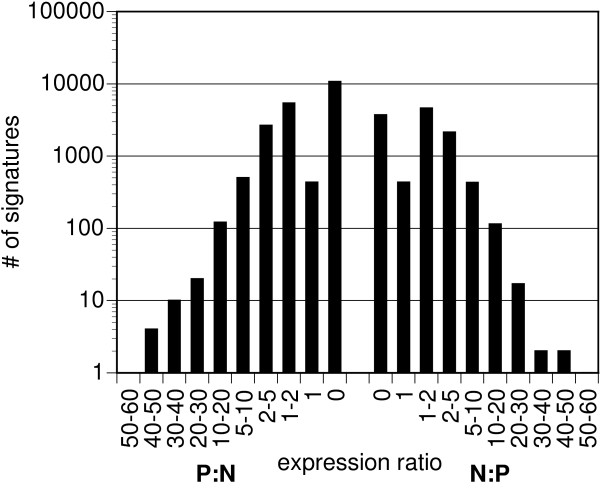
**Comparison of expression for all of the *A. fundyense *signatures**. The expression ratios compare the abundance of each signature between the two libraries. Bars indicate the number of signatures with an expression ratio within the stated range. Expression ratios were calculated to be ≥1, so that signatures that are more highly expressed in the P/40 library are to the left (P:N) whereas those with greater expression in the N/40 library are to the right (N:P). The exceptions are signatures with ratio = 1, these are repeated on the two sides of the figure, and those with ratio = 0.

For those signatures which were present in both libraries, their relative abundance between the two conditions – the expression ratio – varied widely, from 1 to greater than 50 (Figure 3). Almost half of all signatures (46.8%) were condition-specific, in that they were found only in one library and absent from the other (expression ratio = 0). Another 33.8% of signatures were constantly expressed, defined by Meyers et al. (2004) [24] as those signatures with a summed abundance within a two-fold range (expression ratio <2). Only 19.4% of all signatures (6021 signatures) were found in both libraries and showed a two-fold or greater difference in expression. Of these, 15.5% had expression ratios between 2 and 5, and only a small percentage of signatures, 4%, showed more than a five-fold difference in expression level between the two libraries.

**Figure 4:**
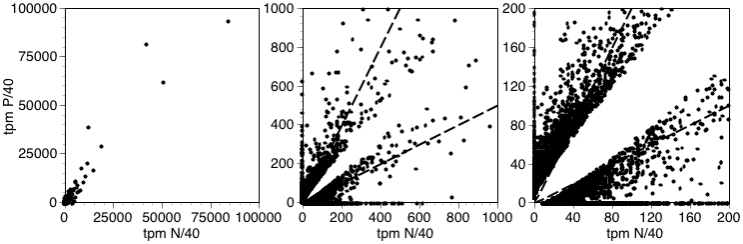
**Paired abundance data for all signatures with differential expression significant at p < 0.05**. Each data point corresponds to a signature with expression that is significantly different between the N/40 and P/40 libraries at p < 0.05. Data are plotted as signature expression in the P/40 library (x-axis) vs. expression in the N/40 library (y-axis). The axis scale decreases from left to right panels to show detail. Dashed line is the line of constant expression, i.e. the point at which the expression ratio is two-fold different between the two conditions.

Signature abundance was also compared statistically between the two libraries using a Z-test [25], with the resulting statistical significance expressed as a p-value. The expression of 11,037 signatures was significantly different at p < 0.05. Of those 11,037 signatures, approximately half (5978) remained significant at p < 0.01, and at p < 0.001 only 3056 signatures showed statistically significant differences in expression (Table 2, Figure 4). Despite having statistical support, approximately 6% of the signatures with significant differences in expression would nonetheless be considered constantly expressed, exhibiting expression ratios < 2 (Table 2, Figure 4). The majority of differentially expressed signatures (ratio ≥2) are those that are condition-specific, i.e. their expression is zero in one of the libraries. About 30% of these signatures at each significance level are common to both libraries.

### RACE amplification using MPSS signatures

We synthesized oligonucleotide primers matching 45 of the MPSS signatures, in order to generate longer fragments via PCR for sequencing and potential transcript identification. This strategy is similar to that described as RAST-PCR using SAGE tags [26], and as GLGI-MPSS using MPSS tags [27], and also to the widely used rapid amplification of cDNA ends (3' RACE). For RACE amplification, we chose 5 types of signatures: the most highly expressed signatures found *only *in the N/40 library (tpm_P _= 0), the most highly expressed signatures found *only *in the P/40 library (tpm_N _= 0), signatures with the greatest relative expression in the N/40 library (tpm_N_>>tpm_P_), signatures with the greatest relative expression in the P/40 library (tpm_P_>>tpm_N_), and signatures that were very highly expressed in both libraries. These 45 signatures exhibited tpm values ranging from 212 to 90502.

Two separate rounds of RACE amplification were performed, with 22 out of the 45 signatures (49%) generating a product in one or both rounds. Product sizes ranged from 106 to 531 base pairs, exclusive of the poly(A) tract (Additional File 1). Seven reactions produced multiple amplicons that exhibited widely differing database matches and thus no definitive identification could be made. Of the remaining 15 signatures, all produced a single product as assessed by gel electrophoresis. Sequences of all except 2 of the 15 exhibited high similarity, at the nucleotide level, to ESTs recently collected from *Alexandrium tamarense *[28]. Further identification was achieved through translated searches of the GenBank database; 12 RACE products matched known dinoflagellate genes, one had no similarities to known proteins, and two showed homology to non-dinoflagellate genes.

Sequence variants of only three known dinoflagellate genes accounted for more than half of the signatures that could be identified (9 of 15). Four of the 15 signatures matched the luciferin-binding protein gene (*lbp*) of the dinoflagellate *Lingulodinium polyedrum*. These include the second and ninth most abundant signatures overall; all four signatures are more abundant in the P/40 library. Four of the 15 signatures matched the histone-like protein genes (*hlp*) known from the dinoflagellates *Crypthecodinium cohnii *and *Lingulodinium polyedrum*. These RACE sequences are also more distantly related to the histone-like protein sequence (*HAf*) identified by Taroncher-Oldenburg and Anderson (2000) [12] in this same organism, sharing homology only in the central domain of the protein. All four of the signatures matching histone-like protein transcripts are more highly expressed in the P/40 library; one of them is the third most abundant signature and the other three are unique to the P/40 library. One of the 15 signatures produced a RACE product that matched the S-adenosyl-homocysteine hydrolase gene (*SAHH*) first identified in *A. fundyense *by Taroncher-Oldenburg and Anderson (2000) [12]. This signature was more highly expressed in the N/40 library and was derived from an upstream DpnII site. The signature including the downstream 3'-end DpnII site matched one of the RACE primers that did not produce a product (the product size would have been 32 bp). This signature was the fifth most abundant signature overall.

The remaining six signatures that were identified each mapped to a different gene. One of these signatures was unique to the P/40 library and showed homology to the ribonucleoside-diphosphate reductase gene of *Arabidopsis thaliana*. Two of the six signatures were moderately expressed and more abundant in the P/40 library; these showed homology to a light-harvesting protein gene from the dinoflagellate *Heterocapsa triquetra *and the cytochrome b gene of the dinoflagellate *Pfiesteria piscicida*. The other three signatures of the six were expressed at low levels in only the N/40 library. These signatures were homologous to the dinoflagellate peridinin-chlorophyll *a*-binding protein, a seed storage protein, and an *A. tamarense *EST.

For most of the signatures, multiple sequence variants were observed amongst the multiple clones of each product that were sequenced. For example, the RACE product generated by signature #49 appeared as a single band on an agarose gel, was cloned and 6 clones were sequenced. All 6 of the sequences matched *lbp*, but the sequences themselves varied in length from 243 to 261 bases, and contained sequence variations ranging from 1–3 indels to 1–3 base pair changes. Thus, in the RACE reactions, we observed both multiple signatures mapping to the same gene, as well as multiple sequence variants of one gene carrying the same signature.

## Discussion

### *Alexandrium *signature content

We have utilized MPSS to examine gene expression in *Alexandrium *cells grown under widely differing physiological conditions, where both nutrient status and toxicity vary. The results of this analysis provide a global view of the transcriptome and its regulation in these unusual single-celled eukaryotes. The number of unique signatures in *Alexandrium *is quite comparable to the values observed in humans and *Arabidopsis*, two other eukaryotes that have been analyzed using MPSS: ~20,000–27,000 signatures per library in *Alexandrium*, compared to ~14,000–45,000 in humans and ~11,000–25,000 in *Arabidopsis *(Table 3) [24,29]. Thus, if the MPSS signature number provides a "rough first estimate of the complexity of gene expression" [30], *Alexandrium *exhibits significant transcriptional complexity, on par with multicellular eukaryotes.

Because dinoflagellates contain so much cellular DNA, yet so little is known about their gene content and organization, it may be tempting to make inferences about gene number based upon signature number. However, even the few paired data available on the number of genes and MPSS signatures (Table 3) show the relationship between the two values to be unpredictable. Studies that have matched expression tags (from both SAGE and MPSS) to their corresponding transcripts from genome and EST sequencing projects (e.g. [29-33]) have revealed a number of reasons why each unique signature does not necessarily correspond to a unique chromosomal gene. Some signatures arise from non-chromosomal genes, primarily mitochondrial or ribosomal transcripts, or from sequencing error. A single gene may produce more than one signature sequence due to alternative splicing, alternative 3' termination and polyadenylation, or cleavage at an upstream restriction site on different mRNA copies. Signatures may be derived from antisense transcripts. Each gene may not produce a unique signature because the transcript may not contain the restriction site, or the site may be too close to the polyA tail to produce a meaningful signature. Two (or more) transcripts may also share the same signature sequence by chance alone. Many of these processes are operating in dinoflagellates, *Arabidopsis *and humans. However, alternative splicing, which contributes to transcriptome diversification, is thought to occur at a much lower rate in unicellular eukaryotes than in multicellular eukaryotes [34]. Nonetheless, for all of these reasons it is problematic to make inferences about gene content based solely upon MPSS signature numbers.

### Signature abundance distribution and expression in *Alexandrium*

The abundance distribution of *Alexandrium *signatures is quite typical of other eukaryotes examined. The vast majority of the signatures (>90%) are present at less than 0.01% abundance (100 tpm), indicating that most genes are expressed at very low levels. If we assume that a cell contains an average of 300,000 mRNA molecules [35], this corresponds to less than 30 copies per cell. In eukaryotes, the vast majority (>90%) of mRNA sequences are present at less than 9 copies per cell, with very few sequences present in a high abundance class that constitutes about one-fifth of the cellular RNA [36]. The *Alexandrium *signature abundance data show this same classic profile, and other recent work using SAGE and MPSS has confirms the generality of this abundance pattern. Zhang et al. (1997) [33] found that 86% of transcripts identified via SAGE were present at less than 5 copies per cell, and only 0.11% of all transcripts were found at >500 copies per cell. MPSS analysis of cultured human cell lines showed only 7 signatures with greater than 10,000 tpm abundance, with ~90% of signatures present at less than 100 tpm [30]. In a variety of *Arabidopsis *tissues analyzed by MPSS, more than two-thirds of signatures are present at less than 100 tpm [31].

The extent of expression regulation also shows similarities between *Alexandrium *and *Arabidopsis*, an organism that has been subject to extensive MPSS analysis [24,31]. In both organisms, about half of all signatures are shared between conditions, and roughly half of those are constantly expressed. Of the shared genes that are differentially regulated, the expression ratios range from 2 to almost 60. These data demonstrate quite clearly that dinoflagellates commonly employ transcriptional regulation of their genes. Even at the most stringent significance values used in Table 2, approximately 10% of total signatures show differential regulation. This is about three times higher than that observed in microarray analyses of *Pyrocystis lunula *[16,17], where approximately 3% of their targets showed expression differences. The difference in the magnitude of differential expression is likely due to multiple factors. The *Pyrocystis *studies used short-term (hours) exposures to their experimental conditions versus the longer (days) exposure to nutrient stress in this study, and the proportion of genes regulated on these varying time scales may be very different. In addition, the sampling depth of MPSS detects more low copy number genes and smaller expression differences, which would contribute to the greater number of differentially expressed transcripts in the MPSS data.

### RACE analysis of MPSS signatures

The abundance and distribution of the *Alexandrium *signatures is very similar to that of other eukaryotes that have been analyzed. The results of the RACE analysis of MPSS signatures, however, suggest a mechanism that may be more common in dinoflagellates and would contribute to the transcriptional complexity of *Alexandrium*. We hypothesize that dinoflagellates exhibit an increased tendency for genes to occur in multiple copies such as long tandem repeats. This could easily increase signature diversity if the repeats are not exact copies of one another, but instead contain sequence differences at or near their 3' restriction sites. This is evident from the RACE results, where only three dinoflagellate genes are responsible for 9 of the mapped signatures – luciferin-binding protein, SAHH and histone-like protein. In addition, many of the signatures produced multiple sequence variants amongst the cloned RACE products, indicating the presence of yet more copies of the gene in question. For example, the four MPSS signatures that matched *lbp *comprised 24 different sequence variants in total.

The MPSS and RACE data provide a global view of a phenomenon that is becoming increasingly apparent in the literature of dinoflagellate gene content and regulation: the presence of widespread gene duplication in these organisms. One of the earliest descriptions of a cloned gene in dinoflagellates – the *lbp *of *Lingulodinium polyedrum *– reported the presence of ~1000 copies of this gene in the genome [9]. The gene encoding the peridinin-chlorophyll *a*-binding protein (*pcp*) of this same species was documented to occur as ~5000 copies arranged in long tandem repeats [8], making *pcp *one of the most highly repeated protein-coding genes ever reported. Tandem repeat organization of ~100 copies has been described for the luciferase gene (*lcf*) of *L. polyedrum *[10], and ~30 copies of a cAMP-dependent protein kinase gene have been found in this organism [37]. The ribulose-1,5-bisphosphate carboxylase-oxygenase (RuBisCO) gene in another species of dinoflagellate, *Prorocentrum minimum*, also exists in multiple copies but with different gene organization. The RuBisCO genes are arranged in about 37 transcribed units, each containing four copies of the coding region [38]. Dinoflagellate mitochondrial genes exhibit similar sequence variation; the genes for cytochrome oxidase subunit I (*cox1*) and apocytochrome *b *(*cob*) occur as multiple copies in several species [39,40]. The *cob*, *cox*1 and *cox*3 genes may exist as polyadenylated yet random fragments in the mitochondria, sometimes with two to four short DNA fragments, either from the same gene or different genes, attached together and co-translated [41].

The growing number of expressed sequence tag (EST) collections for a variety of dinoflagellate species also shows evidence of multicopy genes. In the dinoflagellate *Karenia brevis*, 40% of the EST gene clusters showed single nucleotide polymorphisms (SNPs), indicating the presence of multiple copies of those genes [42]. SNPs have been observed at a similar rate in EST libraries from both *Amphidinium carterae *and *L. polyedrum *[43]. A collection of 6723 unique EST sequences has been generated for *Alexandrium tamarense*, a member of the same species complex as *A. fundyense *[28]. Clustering of their ESTs revealed multiple sequences for *hlp*, *lbp*, *pcp*, ATP synthase, light harvesting protein, RuBisCO, cytochrome c_6_, elongation factor 1-alpha, as well as several unknown and potentially dinoflagellate-specific proteins.

While EST and other sequence data provide ample support for the duplication of multiple genes in dinoflagellates, it does not appear that dinoflagellates have simply duplicated their entire genome. Studies of DNA reassociation kinetics in dinoflagellates [44-46] indicate that their genomes contain roughly 50% repetitive DNA, which is commensurate with eukaryotes in general and argues against polyploidy or polyteny. Furthermore, population genetic studies of dinoflagellates using microsatellite markers have revealed only single alleles, supporting their status as haploid during vegetative growth [47,48]. The presence of multiple copies of many genes does provide some explanation for the large genomes of dinoflagellates, where DNA content ranges from approximately 3 pg of DNA·cell-1 to more than 200 pg·cell-1 [3,4].

## Conclusion

The MPSS data indicate that *Alexandrium *exhibits significant transcriptional complexity, comparable to humans and *Arabidopsis*. Furthermore, dinoflagellates seem to be quite similar to multicellular eukaryotes in terms of signature abundance, distribution, and expression. This includes the observation that expression of a significant number of signatures are differentially regulated, whereas previous studies of dinoflagellate gene regulation implicated translational control as the primary mechanism. The results of signature mapping via RACE add to the growing body of evidence for widespread gene duplication in dinoflagellates, which would contribute to the transcriptional complexity of this organism. Furthermore, extensive duplication of many genes does provide one mechanism for the expansion of the dinoflagellate genome without invoking wholesale genome duplication, although it is unlikely that gene duplication is responsible for the total DNA content of dinoflagellates. The question still remains, however, as to the underlying mechanism for the expansion of individual genes in dinoflagellates.

## Methods

### Isolates and culture conditions

*Alexandrium fundyense *CA28 is a clonal, toxin-producing strain isolated from the Gulf of Maine, Massachusetts, USA. Culture medium consisted of 0.45 μm-filtered natural seawater (Vineyard Sound, MA, salinity 31‰) enriched with F/2 nutrients [49]. Cultures were grown at 20°C on a 14:10 light:dark cycle with cool-white fluorescent illumination of approximately 150 μE·m^-2^·s^-1^. Starter cultures were grown to mid-log phase in one liter of medium. Cells from starter cultures were quantified microscopically and used to inoculate 18 L carboy cultures to a beginning density of 200 cells·mL^-1^. In one carboy, the added nitrate concentration of the medium was reduced to 5% of F/2 level (N/40), while all other nutrients remained the same. Similarly, a second carboy contained reduced-phosphate medium (5% of F/2 = P/40)

### Measurement of cell density, nutrient concentrations and toxin content

At the beginning of each light period, a sample was aseptically withdrawn from each carboy. Triplicate 1 mL subsamples were preserved in Utermohl's iodine solution for cell counts [50]. Cell density was determined daily by counting at least 200 cells from each preserved subsample. A 30 mL subsample was filtered through a 25 mm diameter combusted GF/F filter and the filtrate was stored at -20°C for spectrophotometric determination of nitrate and phosphate concentrations.

### RNA preparation

Cells were harvested for RNA isolation when the growth rate slowed, at the transition between log and stationary growth phases. Also at that time, one liter of culture was transferred to a sterile flask for continued monitoring. The remaining culture volume was processed in one liter portions by passage through a 20 μm nitex sieve. The resulting cell concentrate was washed from the sieve, pelleted by centrifugation and resuspended in one mL of RNAWiz (Ambion, Inc.) then immediately frozen in liquid nitrogen.

Approximately 1 × 10^7 ^cells were used for RNA extraction. Cell suspensions were removed from liquid nitrogen and thawed at room temperature. After the addition of 0.5 mm zirconium beads, they were processed by 3, 50-second cycles in a mini-beadbeater (BioSpec Products), and total RNA was isolated from the cell lysate following the manufacturer's protocol. Total RNA was quantified by spectrophotometer and its integrity was assessed by agarose gel electrophoresis. Poly(A)^+ ^RNA was purified by two rounds of selection using the Poly(A)Pure kit (Ambion, Inc.) according to the manufacturer's directions. The poly(A)^+ ^RNA was quantified spectrophotometrically, and 2 μg was sent to Solexa, Inc. for Massively Parallel Signature Sequencing.

### Massively Parallel Signature Sequencing (MPSS)

The mRNA was processed through the MPSS protocol essentially as described in Brenner et al. (2000) [20]. Briefly, the mRNA was reverse transcribed to cDNA. The cDNA was digested with DpnII and the 21 bases adjacent to the 3'-most DpnII site was cloned into a vector. The resulting library was PCR amplified and loaded onto microbeads. About 1.6 million microbeads was loaded into each flow cell and the sequence of the 17 bp at the 5' end of each fragment was determined via a series of enzymatic reactions, described in detail in Brenner et al. (2000)[20]. More than 1 million **sequences **were obtained from each sample. The occurrence of each specific non-redundant 17 bp sequence, termed a **signature**, is summed across all sequences obtained from a library. The abundance of each signature is then normalized to one million (transcripts per million, tpm) for the purpose of comparison between samples. Signature abundance is the N/40 library is herein referred to as tpm_N_, with tpm_P _designating the abundance in the P/40 library.

### Analysis of MPSS data

Two filters were applied to the complete set of unique signatures derived from the N/40 and P/40 samples in order to remove signatures that may arise from errors in the MPSS procedure (these filters are discussed in detail in [24]). The first filter – the "reliability filter" – removes any signature that is observed in only one sequencing run across all libraries, with the aim of eliminating signatures that result from technical problems specific to a single sequencing run. The second filter – the "significance" filter – selects for only those signatures that are found at 4 tpm or above in at least one library. The goal of this filter is to remove signatures that are consistently present at background levels. A cut-off of 4 tpm as opposed to 3 tpm was chosen because, in the *Arabidopsis *analysis, 4 tpm was found to be different from 0 tpm at p < 0.05, and 1, 2 or 3 tpm was not significantly different from 0 tpm (p > 0.05) [31]. For consistency, the human MPSS data [29] were downloaded from the authors' website [51] and analyzed using these two filters, as the data analysis presented in the paper used a significance cut-off of 3 tpm.

### Generation and analysis of 3' cDNA fragments using MPSS signatures

A procedure similar to 3' rapid amplification of cDNA ends (RACE) was used to obtain 3' cDNA fragments for identification of the transcripts from which the signatures were derived (thus, they are referred to hereafter in the text as "RACE" products, reactions, etc). mRNA was purified as described above, from additional aliquots of the cells used for MPSS. Reverse transcription was performed using ThermoScript RT (Invitrogen Corp.) according to the manufacturer's instructions and an anchored oligo-dT primer incorporating a priming site for later PCR amplification (3RACEdT, 5'-AAG CAG TGG TAT CAA CGC AGA GTA CT_30_VN-3' where V = A/G/C). The resulting cDNA was PCR amplified using the 17 bp MPSS signature sequence as the 5' or forward primer and the synthetic primer sequence as the reverse primer (3RACE, 5'-AAG CAG TGG TAT CAA CGC AGA GTA C-3'). PCR reactions were performed in a final volume of 25 μL containing 1× PCR buffer, 100 μM dNTPs, 0.4 μM each primer, 1.5 μL of cDNA and 2.5 U of Taq polymerase (New England Biolabs). Amplification consisted of an initial hold at 95°C for 2 min., followed by 45 cycles at 95°C for 30 sec., annealing temp. for 45 sec. and 72°C for 1 min., followed by a final 10 min. incubation at 72°C. Because the annealing temperatures of the different MPSS signature primers varied, PCR was performed in a gradient PCR cycler, and reaction tubes were placed in the block positions corresponding to a temperature of 5°C below the calculated T_m _of the MPSS signature primer.

### Cloning and sequencing

The presence of RACE products was verified by agarose gel electrophoresis of 10% of the total reaction volume. For all reactions in which only a single amplicon was observed, the remaining reaction volume was purified using the Qia PCR cleanup kit (Qiagen Inc.), and the purified product was cloned into the pCRII-TOPO vector (Invitrogen Corp.). Several clones from each of the RACE products (usually 6–8) were sequenced in both directions, using vector primers and Big Dye Terminator sequencing chemistry v3.0 (Applied Biosystems). Sequencing reactions were visualized on an ABI 3730xl capillary sequencer (Applied Biosystems) and edited using Sequencher (GeneCodes Corp.). Sequences were compared to the GenBank nr and est databases using the tblastx algorithm, to determine similarity to known gene sequences. RACE products are deposited in GenBank under the sequential accession numbers DY241874-DY241888.

## Authors' contributions

DLE performed the experimental work and drafted the manuscript. DMA participated in study design and manuscript preparation. All authors read and approved the final manuscript.

## Supplementary Material

Additional File 1 Results of RACE amplifications using MPSS signatures. landscape formatted table containing text detailing the results of sequencing analyses. Click here for file

## Figures and Tables

**Table 1:** Summary of library characteristics

	**N-starved cells**	**P-starved cells**
**Total sequences collected**	2,259,219	1,501,972
**Total signatures**	44,779	27,722
**Reliable, significant signatures**	27,217	20,161
**Signatures unique to library**	10,756	3,700

**Table 2:** Statistical differences in signature expression

**Significance level**	**Number of signatures with statistically significant expression**	**Number of statistically significant signatures with expression ratio ≥2**
p < 0.05	11,037	10,284^a ^(3053^b^)
p < 0.01	5978	5624 (1720)
p < 0.001	3056	2872 (868)

^a ^All signatures that have expression ratio ≥2

^b ^Only those signatures common to both libraries

**Table 3:** MPSS data, DNA content and predicted gene number for various organisms

**Organism**	**MPSS Signatures per Cell Type**	**Total Signature Number**	**Average Signatures per Cell Type**	**Genome size**	**Predicted Gene Number**
*H. sapiens*	13,510 – 44,634	181,415^a^	29,285	3200 Mb	20,000–25,000^d^
*Arabidopsis thaliana*	11,374 – 25,229	87,705^b^	19,750	125 Mb	~30,000^e^
*A. fundyense*	20,161 – 27,217	30,917	23,689	ca. 96,000 Mb^c^	n/a

^a ^Data from Jongeneel et al. (2005) [29], filtered as described in Materials and Methods

^b ^Meyers et al. (2004) [24]

^c ^based on values for *A. tamarense*, LaJeunesse et al. (2005) [3]

^d ^International Human Genome Sequencing Consortium (2004) [52]

^e ^TIGR Arabidopsis genome annotation v5 [53].
