# Supplementary material for: Global transcriptional profiling of the toxic dinoflagellate Alexandrium fundyense using Massively Parallel Signature Sequencing
Source: BMC Genomics. 2006 Apr 25;7:88. doi: 10.1186/1471-2164-7-88 (PMC1473201; doi:10.1186/1471-2164-7-88)
Supplement: Additional File 1 — Results of RACE amplifications using MPSS signatures. landscape formatted table containing text detailing the results of sequencing analyses. [file 1471-2164-7-88-S1.pdf]

Results of RACE amplifications using MPSS signatures

#	MPSS signature	tpm_N	tpm_P	bp	Best match (nucleotide BLAST) / Best match (translated BLAST)	% id / E val	Accession # of match
42	GATCACCTACCGTCCGG	50667	62195	317	*Lingulodinium polyedrum* luciferin-binding protein mRNA<br>*A. tamarense* EST UI-D-GC1-aaq-b-08-0-UI	95	CK782963
					ND[a]		
49	GATCAGACCCCCTGCGA	9609	10532	253	Same as previous signature		
22	GATCACCTACCGACCGG	199	3360	325	Same as previous signature		
15	GATCACCTACCGACCGT	0	274	315	Same as previous signature		
34	GATCCATGCGGTCTTTG	42044	81751	106	*A. tamarense* EST UI-D-GC1-aap-d-10-0-UI	94	CV554063
					Lingulodinium polyedrum histone-like protein mRNA	3e-34	AF482694
11	GATCCATGCGGTCTTCG	0	464	109	Same as previous signature		
12	GATCCATGCGGTATTTG	0	373	109	Same as previous signature		
17	GATCCATGCGCTCTTTG	0	260	108	Same as previous signature		
35	GATCGTACAGGCAACAT	2209	282	531	*Alexandrium fundyense* S-adenosyl-homocysteine hydrolase like protein mRNA<br>*A. tamarense* EST UI-D-GC0-aae-p-02-0-UI	99<br>95	AF105295<br>CF947581
					ND[a]		
45	GATCCTGTAGCGATGTG	14664	16688	-	Same as previous signature; the #45 RACE sequence maps to a downstream GATC site on the #35 sequence		
14	GATCTCATTGCAGGGGT	0	279	320	*A. tamarense* EST UI-D-GC1-aau-b-17-0-UI	89	CK783780
					Arabidopsis thaliana putative ribonucleoside-diphosphate reductase small chain	2e-5	BT004167
26	GATCGCCATGTTCGCGG	2059	6153	109	*A. tamarense* EST UI-D-GC1-abf-c-02-0-UI	90	CK785886
					Heterocapsa triquetra chloroplast light harvesting complex protein[b]	2e-22	AY826911
23	GATCCTTGTGAATTAAC	671	2930	250	*Lingulodinium polyedrum* EST	95	BP742407
					Pfiesteria piscicida mitochondrial cytochrome b	2e-26	AF357521
7	GATCTGATTGCGTGGGC	302	0	228	*A. tamarense* EST UI-D-GC1-aaq-a-01-0-UI	93	CV555282
					Hordeum vulgare DNA for B-Hordein seed storage protein[b]	4e-9	X53690
9	GATCAAGTCAAGTATGC	281	0	112	*A. tamarense* EST UI-D-GC1-aba-h-15-0-UI	93	CK784543
					none		
4	GATCGCATCCATGCCTA	354	0	401	*Lingulodinium polyedrum* EST	89	CD809716
					A. tamarense pcp	9e-91	AB126029

[a] not determined because a match was made via nucleotide search

[b] translated blast match was made to the best matching EST, not to the RACE product itself.
